# Incidental Median Nerve Injury in the Hand by a High-Speed Drill

**DOI:** 10.7759/cureus.21243

**Published:** 2022-01-14

**Authors:** Georgi P Georgiev

**Affiliations:** 1 Orthopaedics and Traumatology, University Hospital Queen Giovanna - ISUL, Sofia, BGR

**Keywords:** high-speed drill, injury, hand, suture, median nerve

## Abstract

Traumatic injuries of the peripheral nerves can be easily overlooked in the emergency department. In these cases, extensive knowledge of anatomy, a high index of suspicion and correct evaluation of neurological status could maintain the proper diagnosis.

Median nerve injury in the region of the hand is commonly due to glass material. However, extremely rare, other causes of injury, such as pieces of wood, acupuncture needle-fracture bodies, metallic foreign bodies, and migration of K-wires, have been reported. In the current report, a case of median nerve injury in a 35-year-old man treated with a high-speed drill was reported to my knowledge for the first time in the current literature. Early and correct diagnosis for nerve injury is crucial for improved functional outcomes.

## Introduction

Peripheral nerve injuries are still a serious problem in current medical practice. They are commonly observed after motor vehicle accidents, penetrating trauma, or falls. Direct trauma of the hand and nerve injury could have dramatic effects on patient quality of life and cause severe consequences for hand function. Therefore, initial evaluation by “front line” physicians is crucial for step-by-step approaches, including possible bone or neurovascular injury [1].

Median nerve (MN) injury in the region of the hand is commonly due to glass material [2]. However, extremely rare, other causes of injury, such as pieces of wood [3], acupuncture needle-fracture bodies [4], metallic foreign bodies [2], and migration of K-wires [5], have been reported. To date, there is a lack of reports of MN injury in the hand by a high-speed drill.

## Case presentation

**Figure 1:**
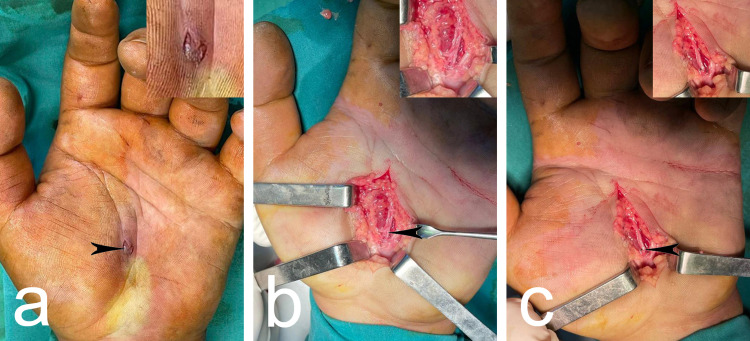
Preoperative photograph presenting the puncture/entry wound (arrowhead) (a); intraoperative photograph presenting the lesion (arrowhead) (b); intraoperative photograph after suture (arrowhead) (c).

Herein, we present a 35-year-old man brought to our department after trauma with a high-speed drill during installation of aluminum siding, with a complaint of a wound and lack of sensitivity of the third finger of the left hand. The used drill was 6 mm in diameter. After cleaning the wound and tetanus antitoxin injection, roentgenography of the hand was performed. The anteroposterior and oblique views did not show any bony injuries or fractures. During the physical examination, a puncture/entry wound, 7 x 5 mm in size, near the thenar eminence, localized between the two thenar creases distal to the carpal tunnel, approximately 3.5 cm distal to the distal wrist crease and approximately 2.5 cm proximal to the proximal palmar crease was observed (Figure 1a). There were no signs of reduced sensation, except in the third finger; no flexor tendons were affected. Under anesthesia block and proximal tourniquet, the patient underwent surgical exploration. The wound was extended proximally at the level of the end of the carpal tunnel and distally to the proximal palmar crease. In depth, injury to the sensory branch of the MN that passed along the center ray of the hand was detected (Figure 1b). No other damages of the MN were observed. The branch was adapted and sutured by 5/0 ethilon sutures (Figure 1c).

After obtaining hemostasis with bipolar coagulation, the skin was closed with 3/0 ethilon, and a cast in slight wrist flexion was applied. A triple antibiotic intravenous treatment with ceftriaxone, amikacin and metronidazole for three days was performed, followed by oral administration of levofloxacin for five days. Medical nerve regeneration therapy with curcumin (300 mg/d), lecithin (600 mg/d), Mangifera indica (200 mg/d), vitamin B complex (benfotiamine - 80 mg/d, pyridoxine hydrochloride - 180 mg/d, cyanocobalamin - 500 μg/d) and acetyl-L-carnitine (1,000 mg/d) was applied for two months. The stitches were removed 14 days after surgery; the splint was removed 25 days after surgery, and physiotherapy was applied to reduce swelling and scar management. After six months, the patient showed complete resolution of impaired sensation symptoms.

## Discussion

Penetrating injuries by foreign bodies in the hand are not uncommon and are usually not combined with damage to the peripheral nerve. However, there is a high level of suspicious need in wounds around the anatomical area of the nerves to eliminate the omission of the nerve lesion, especially in cases of any doubt regarding nerve injury. In all penetrating injuries, a neurological status distal to the injury should be established. In cases of the potential nerve, lesion surgery is mandatory [6,7].

After an extensive review of PubMed, only five cases of uncommon penetrated injury of the MN injury were found, but none of them were similar to the presented case. First, in 1972, El-Adwar presented a case of MN compression in a 48-year-old man due to a piece of wood falling on a heap of grass [3]. Later, Southworth and Hartwig described a case of a 77-year-old woman operated on for MN neuropathy due to a fractured acupuncture needle within the nerve [4]. Faithfull and Petchell presented a case of occult MN injury by a metallic foreign body in a 40-year-old male after an injury during work with a coldchisel and hammer [2]. Jou and Lai described a case of acute MN injury in a 29-year-old man due to migration of a K-wire after the operation due to untreated scaphoid nonunion combined with dorsal perilunate dislocation [5]. Rainer et al. presented a case of a 56-year-old woman with reduced sensitivity in the index finger after unnoticed penetrating injury of the right wrist by a piece of wood, which subsequently injured the MN [8].

The presence of different foreign bodies in the hand could be a real problem for proper diagnosis and treatment, especially in cases when a clear history of a penetrated agent is missed. Clinicians should keep in mind the possible existence of a retained foreign body in cases of persistent infection of the wound or neurological symptoms [8]. In these cases, radiography needs to be performed. Additional ultrasound, computed tomography, or magnetic resonance imaging could also help in a proper diagnosis of nerve injury or detect the correct location of the foreign body in relation to the MN [8].

Operative treatment of peripheral nerve injuries is a real challenge for surgeons [9]. For early diagnosis and treatment, it is mandatory to know the neurobiological mechanisms and anatomy. In cases of missing, delayed, or inappropriate diagnosis and treatment, a worse functional outcome will be established. The successful treatment of these injuries depends on two excessive factors: the qualification of the hand surgeon and physiotherapist [9].

Immediate nerve repair is the best treatment. The penetrating wound needs to be widely exposed; thereafter, careful nerve dissection and primary repair should be performed. Plaster immobilization for up to three weeks for digital nerve repair is recommended [9].

## Conclusions

In conclusion, the reported mechanism of the injury and the affected anatomical area should attract attention to possible MN damage. The diagnosis is based on thorough anamnesis and precise neurological evaluation. The treatment includes surgical exploration with excision of the damaged tissues, detection of the nerve lesion, precise adaptation and restoration of the nerve end by end and antibiotic treatment and medical nerve regeneration therapy. Although these injuries are not life threatening, when overlooked, the consequences for the hand could be severe.
